# The Role of 18F-FDG PET/CT in the Evaluation of Gastric Cancer Recurrence

**DOI:** 10.4274/mirt.83803

**Published:** 2014-10-05

**Authors:** Hakan Cayvarlı, Recep Bekiş, Tülay Akman, Deniz Altun

**Affiliations:** 1 Ordu State Hospital, Clinic of Nuclear Medicine, Ordu, Turkey; 2 Dokuz Eylül University Faculty of Medicine, Department of Nuclear Medicine, İzmir, Turkey; 3 Tepecik Training and Research Hospital, Clinic of Medical Oncology, İzmir, Turkey; 4 Dokuz Eylül University Faculty of Medicine, Department of Public Health, İzmir, Turkey

**Keywords:** Gastric cancer, Positron-emission tomography, 18F-FDG, disease management

## Abstract

**Objective:** F-18-fluorodeoxyglucose positron emission tomography/computed tomography (18F-FDG PET/CT) has been widely used for staging, re-staging and for monitoring therapy-induced changes and response to therapy in patients with various types of cancer, but its utilization for gastric cancer has been limited. This study aimed to assess the diagnostic performance of 18F-FDG PET/CT for detecting recurrence in gastric cancer patients with radiologic or clinical suspicion of recurrence and its clinical impact on making decision.

**Methods:** We performed a retrospective review of 130 consecutive patients who underwent PET/CT scans for post-treatment surveillance of gastric cancer between January 2008 and March 2012. The mean time between the initial diagnosis of gastric cancer and PET/CT studies was 44 weeks with a median of 18 weeks. The number and site of positive FDG uptake were analyzed and correlated with the final diagnosis by calculating the diagnostic values. We evaluated the diagnostic accuracy of PET/CT for detecting the recurrence in terms of whether or not histology had been SRC/musinous adenocarcinoma. The changes in the clinical management of patients were also evaluated according to the results of PET/CT.

**Results:** Of all 130 patients, 91 patients were confirmed to have true recurrence. The sensitivity, specificity, positive predictive value, negative predictive value and the accuracy of PET/CT for diagnosing true recurrence on a per-person basis were 91.2%, 61.5%, 84.6%, 75.0% and 82.3% respectively. Final diagnoses were confirmed histopathologically in 59 (45.4%) of 130 patients and by clinical and radiological follow-up in the remaining 71 (54.6%) patients. In the subgroup with SRC/mucinous adenocarcinoma differentiation of the primary tumor, there was no statistically significant difference in terms of diagnostic accuracy of PET/CT on a per-person basis. In addition, PET/CT results changed the patients’ management in 20 (15%) cases.

**Conclusions:** 18F-FDG PET/CT can provide useful information in discriminating true recurrence in patients with suspected gastric cancer recurrence and may have significant impact on clinical decisions/patient management in a considerable percentage of patients.

## Abstract (tr)

**Amaç:** F-18-florodeoksiglukoz pozitron emisyon tomografisi/bilgisayarlı tomografi (18F-FDG PET/BT) pek çok kanser türünde evrelemede, yeniden evrelemede, tedaviye bağlı değişikliklerin ve tedavi yanıtının değerlendirilmesinde yaygın olarak kullanılmaktadır ancak mide kanserinde kullanımı konusunda yayınlar sınırlı sayıdadır. Bu çalışmada radyolojik ya da klinik olarak rekürrens şüphesi olan mide kanseri hastalarında 18F-FDG PET/BT’nin rekürrensin değerlendirilmesindeki tanısal etkinliğini ve tedavi kararına etkisini saptamayı amaçladık.

**Yöntem:** Mide kanseri tanısı ile tedavi sonrası izlemde olan ve Ocak 2008 ile Mart 2012 tarihleri arasında rekürrens şüphesi ile PET/BT yapılmış 130 hasta geriye dönük olarak değerlendirildi. Hastaların mide kanseri tanısı almaları ile PET/BT çekimleri arasında geçen ortalama süre 44 hafta idi. Patolojik FDG tutulumu izlenen alanların sayı ve bölgeleri incelendi ve kesin tanıları ile karşılaştırılarak PET/BT’nin tanısal etkinliği hesaplandı. Taşlı yüzük hücreli ve müsinöz adenokarsinom histolojik türleri ayrıca değerlendirilerek bu grupta PET/BT’nin tanısal etkinliği incelendi. PET/BT sonuçlarının hastanın klinik takip ve tedavi kararına olan etkisi ayrıca incelendi.

**Bulgular:** Yüz otuz hastanın 91’inde rekürrens saptandı. Hasta bazında değerlendirmede PET/BT’nin duyarlılığı, özgüllüğü, pozitif öngörü değeri, negatif öngörü değeri ve tanısal doğruluğu sırasıyla %91,2, %61,5, %84,6, %75,0 ve %82,3 olarak bulundu. Yüz otuz hastanın 59 tanesinde (%45,4) kesin tanı histopatolojik olarak doğrulanırken, kalan 71 hastada (%54,6) kesin tanıya klinik ya da radyolojik takip neticesinde ulaşıldı. Taşlı yüzük hücreli ve müsinöz adenokarsinom histolojik türleri ile diğer histolojik türler arasında hasta bazında PET/BT’nin tanısal doğruluğu arasında anlamlı bir fark bulunamadı. Ayrıca PET/BT sonuçları doğrultusunda 20 (%15) hastanın önceden planlanan takip/tedavi yöntemi değiştirildi.

**Sonuç:** 18F-FDG PET/BT mide kanseri rekürrensi şüphesi olan hastalarda rekürrensin doğru olarak değerlendirilmesinde faydalı olabilir ve hastaların anlamlı bir kısmında klinisyenin takip/tedavi kararında önemli etkiye sahip olabilir.

## INTRODUCTION

Gastric cancer is the fourth most common cancer worldwide and is the second most common cause of cancer-related deaths (1,2). Radical surgical resection of gastric cancer with lymph node dissection is a considered curative treatment. But its long-term survival is frequently reported as poor. In fact, despite successful surgery, the five year survival rate is approximately 35%, and even with adjuvant chemoradiotherapy in selected patients, the survival rate is 40% (3). After curative surgery, about 80% of the patients die within a short period of time from locoregional recurrence (87%) and/or distant metastasis (30%) (4). The reported recurrence rate after curative surgery ranges between 22% and 60%, increasing with more advanced tumor stage (5,6). It is reported that survival after the diagnosis of recurrent disease was better when recurrence was detected at an asymptomatic stage. Therefore, early detection of recurrence is important in an effort to improve prognosis (7).

The definitive method for diagnosis of gastric cancer recurrence is the pathological confirmation. However, getting adequate tissues is often difficult because either recurred tumor size is very small or it is deeply located or too close to great vessels or organs for needle biopsy. Various methods such as tumor markers, endoscopy or imaging studies can be used to detect gastric cancer recurrence. However each has some limitations: tumor markers cannot localize the recurrent site and endoscopy cannot assign extraluminal recurrence (8). Today the most commonly used imaging method for detection of gastric cancer recurrence is contrast-enhanced computed tomography (CT). It can detect both local recurrence and distant metastasis. But CT has also some limitations on diagnosis of gastric cancer recurrence. Because its diagnostic criteria depends on morphological changes and size measurement but not viability, CT cannot detect the presence of viable tumor tissue and also small lesions like peritoneal implants. In addition, it is difficult to differentiate the post-operational changes from recurrence site (8,9,10,11).

A positron emission tomography (PET) scan is a non-invasive imaging modality that reflects cancer cell metabolism via glucose utilization using fluorodeoxyglucose (18F-FDG) as a tracer (11). Recently positron emission tomography combined with computed tomography (PET/CT) scans are frequently performed for evaluating gastrointestinal tumors; like esophageal and colorectal cancer (12,13). However, at present, limited data is available on the use of FDG PET/CT in gastric cancer recurrence, and the role of 18F-FDG PET/CT scan in detecting gastric cancer recurrence after curative gastrectomy is unclear (2,14,15,16). Enthusiasm for evaluating FDG PET/CT in this type of cancer has probably been tempered by frequent false negative PET findings due to absence of FDG avidity of signet ring cell carcinoma (SRC) and mucinous adenocarcinomas (17).

In clinical practice, it is hard to make treatment decision when gastric cancer recurrence is suspicious in contrast CT but tissue confirmation is difficult. In this case, additional PET/CT can give us more information on detection of recurrence (15). Therefore, the aims of this study were; (1) to evaluate the diagnostic accuracy of PET/CT in diagnosis of gastric cancer recurrence, (2) to evaluate whether the sensitivity of PET/CT for detection of recurrent disease is related to the histological type of the primary (resected) gastric cancer and (3) to assess its clinical impact on decision making. 

## MATERIALS AND METHODS

From January 2008 to March 2013, 130 consecutive PET/CT scans of patients with gastric cancer, who underwent FDG PET/CT scan due to radiologic or clinical suspicion of recurrence during follow-up, were retrospectively analysed after a computerized review of the PET/CT database of our institution. If the patients had repeated PET/CT scans, we only analyzed the results of the first PET/CT scans. Of these 130 patients, 96 were male and 34 were female, with a mean age of 61, ranging from 37 to 84. PET/CT scan indications of the patients were classified into these three following groups: Group 1 (n=112) included patients who were suspected of having recurrence by other imaging modalities such as CT or Magnetic resonance imaging (MRI); Group 2 (n=5) included patients who were suspected of having recurrence because of increase in tumor markers without definite findings on prior imaging modalities or clinical manifestations such as weight loss; and Group 3 (n=13) included patients who were not suspected of having recurrence, but a PET/CT scan was ordered just for follow-up, based on physicians’ request. The mean time between the initial diagnosis of gastric cancer and PET/CT studies was 44 weeks with a median time of 18 weeks and a minimum time of 4 weeks. Patients who were followed-up for less than 3 months after PET/CT scan and patients with proven second malignancy were not included in this study. Patient characteristics are listed in Table 1.

All scans were performed by a PET/CT system (Philips Gemini TOF 3D Mode, Netherlands). In all patients, blood glucose levels were checked, and PET/CT scan was performed if blood glucose level less than 200 mg/dl had been ensured. The patients were asked to fast for at least 6 hours before undergoing PET/CT scan and 370-555 MBq (10-15 mCi) of FDG was administered intravenously 1 hour prior to imaging. For the optimal delineation of bowel structures, 400-600 ml of contrast material diluted with water was ingested 1 hour before PET/CT imaging. CT was performed prior to PET, and the resulting data was used to generate an attenuation correction map for PET. Five-milimeter thick sections were obtained at 80 mA (but adjusted for body thickness) and 120 kV from the skull base to the mid-thigh. All patients were allowed shallow respiration during CT scanning. Next, PET was performed without changing the patient’s position with a 2-minute emission acquisition per imaging level and finally the images were reconstructed. PET image data sets were reconstructed iteratively by applying the CT data for attenuation correction, and coregistered and reconstructed images (5 mm contiguous axial cuts) were displayed on a workstation.

PET/CT images were analyzed by two nuclear medicine physicians. Both readers had knowledge of the clinical findings and of the results of all the available imaging studies. The readers, however, were blinded to the follow-up data. In the event of diagnostic discordance between the readers, a consensus diagnosis was generated. FDG uptake was defined to be positive qualitatively when a focal FDG uptake was higher than the normal biodistribution of background FDG activity. Focal hypermetabolic activity of anostomotic site or remnant stomach was considered as locoregional tumor recurrence. Focal hypermetabolic activity within the liver which was greater than adjacent normal liver tissue was considered as abnormal. Multinodular or diffuse hypermetabolic activities along the intestine or mesentery were considered as findings of peritoneal carcinomatosis. Diffuse mild activity in the intestinal tract was considered as normal physiologic uptake. In addition, to exclude the physiologic uptake, FDG uptake in the bowel was regarded to be positive only when there was wall thickening of the same bowel at simultaneously acquired CT. Any focal activity in the mediastinum which is higher than mediastinal blood pool was regarded as abnormal. PET/CT images were analyzed for the number and site of positive FDG uptake. Standardized uptake values (SUV) of all positive FDG uptakes were measured. For the purpose of statistical analysis, a true-positive lesion was a lesion which was seen on FDG PET/CT images and found to be positive for tumor tissue at histopathological examination or clinical follow-up. A false-positive lesion was a lesion which was seen on FDG PET/CT images but found to be negative for tumor tissue at histopathological examination or clinical follow-up. Moreover, a true-negative lesion was defined when no lesion was seen on FDG PET/CT images and the results of histopathological examination for tumors or clinical follow-up were negative. A false-negative lesion was a lesion that was missed at image analysis but was found to be positive for malignancy at histopathological examination or clinical follow-up. The gold standard consisted in radiological and clinical follow-up or histopathological confirmation. A negative clinical and/or radiological follow-up of at least 3 months starting at the time of the PET/CT scan was required in order to define a lesion as negative. Patient-based classification was performed by considering patients with at least one equivocal or positive PET lesion as positive and all others as negative. If more than one lesion was present in the same patient with discordant diagnostic PET classification, the following rules were used: (a) patients who had at least one true positive PET lesion were classified as true-positive; (b) patients with a false positive and a false negative lesion were classified as false negative. Then, the PET/CT data were correlated with the final diagnosis, by calculating the diagnostic values of PET/CT. The final diagnosis of recurrence was obtained from the results of histopathological examination after surgery, laparotomy or biopsy, and clinical follow-up of at least 3 months. Clinical recurrence was defined as the detection of recurrent disease by imaging modalities like diagnostic CT or MRI within 3 months of the PET/CT scan. Radiologically, recurrence was defined to be present when a suspicious lesion at CT or MRI showed the interval increment in size during follow-up or a suspicious lesion showed the interval decrement in size after radio/chemotherapy. Recurrence detected more than 3 months after the PET/CT scan was interpreted as a new recurrence. All recurrent lesions were classified into 5 categories: 1) locoregional recurrence; the recurrence in remnant stomach or anostomotic site, 2) regional lymph node recurrence; the recurrence in pancreatic, splenic and perigastric lymph nodes along the lesser and greater curvatures, 3) liver; the recurrence of hepatic metastasis form, 4) peritoneal carcinomatosis, and 5) distant metastasis; the recurrence in lymph node or organ except for the liver. Multiple lesions within one category were considered as a one lesion.

Patient and organ based sensitivity, specificity, positive predictive value (PPV), negative predictive value (NPV) and diagnostic accuracy were calculated by using standard statistical operations. Differences between categorical variables in the population were evaluated using Fisher’s exact test. Quantification of tumor metabolic activity was obtained using the SUV normalized to body weight. Mean ± SD of maximum-pixel SUV (SUVmax) of the lesions were calculated. We have also analyzed the diagnostic performance of PET/CT whether or not the histology was SRC/mucinous adenocarcinoma on a per-person and per-lesion basis. The influence of PET/CT on patient management and decision making was retrospectively evaluated together with the clinicians from patients’ records. The analyses were carried out using SPSS 15.0 (SPSS Inc, Chicago, IL, USA). Tests with p value <0,05 were considered as statistically significant. 

This study was designed to be a retrospective analysis based on medical records and was approved by the institutional review board of Dokuz Eylül University Hospital. Informed consent was given by each patient included in this study.

## RESULTS

Of 130 patients evaluated in this study, 91 patients were confirmed to have recurrent disease, and the remaining 39 patients were considered as negative for recurrence according to the final diagnosis. When comparison between the PET/CT findings and final diagnosis was made on a per-person basis; among 91 patients having true recurrence, 83 and 8 patients showed positive and negative FDG uptakes respectively. The remaining 39 patients not having true recurrence showed positive and negative FDG uptakes in 15 and 24 patients, respectively. Therefore; the sensitivity, specificity, PPV, NPV and accuracy of PET/CT for diagnosing true recurrence on a per-person basis were 91.2%, 61.5%, 84.6%, 75.0% and 82.3%, respectively. Final diagnoses were confirmed histopathologically in 59 (45.4%) of 130 patients and by clinical and radiological follow-up in the remaining 71 (54.6%) patients.

On the organ-based analysis, in all of 5 groups, the diagnostic accuracy rates were higher than 75%, which was up to 86% in detecting regional lymph nodes. Sensitivity, specificity, PPV and NPV were also higher than 70%, except for the specificity of detecting locoregional recurrence and NPV of detecting peritoneal carcinomatosis (Table 2).

**Figure 1:**
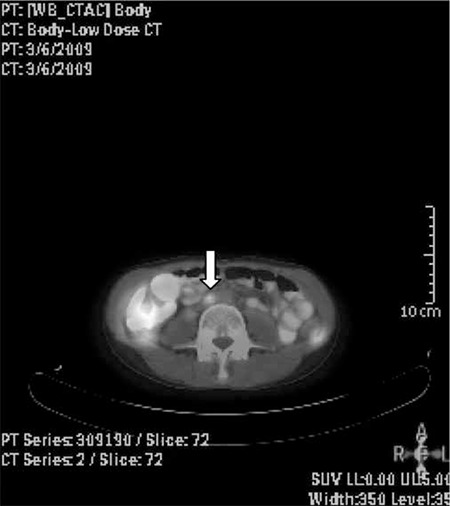
Paraaortic metastatic lymph node (arrow) detected by PET/CT in a patient who was suspected of having recurrence because of increase in tumor markers without definite findings on prior imaging modalities.

When we evaluate distant metastasis group separately, the most common five distant metastasis sites were found as lung, mediastinal lymph nodes, paraaortic lymph nodes, cervical lymph nodes and bones. The diagnostic accuracy values were higher than 75% in all of these sites, which was up to 92% in detecting cervical lymph nodes (Table 3) (Figure 1).

In addition, we have analyzed PET/CT data in terms of whether or not histology had been SRC/musinous adenocarcinoma in initial diagnosis. The sensitivity, specificity, PPV, NPV and accuracy in cases of non SRC/mucinous adenocarcinoma (n=105) were 93.1%, 65.6%, 86.0%, 80.7% and 84.7% respectively, whereas those in cases of SRC/mucinous adenocarcinoma (n=25) were 83.3%, 42.8%, 78.9%, 50.0% and 72.0%, respectively, when comparison was made on a per-person basis. The diagnostic accuracy of PET/CT was higher in the non SRC/mucinous adenocarcinoma group but the difference was not statistically significant. Final diagnoses were confirmed histopathologically in 14 of 25 patients in cases of SRC/mucinous adenocarcinoma and in 45 of 105 patients in cases of non SRC/mucinous adenocarcinoma. We also analyzed PET/CT data in terms of whether or not histology had been SRC/mucinous adenocarcinoma in initial diagnosis on a per-lesion basis. The diagnostic accuracy rates of PET/CT were higher in regional lymph node, peritoneal carcinomatosis, liver and distant metastasis in the non SRC/musinous adenocarcinoma group, and it was higher for locoregional recurrence in the SRC/musinous adenocarcinoma group, but the difference between two groups was not statistically significant except for detection of liver metastasis in the non SRC/musinous adenocarcinoma group (p=0.02) (Table 4).

Overall, therapeutic management was determined by PET/CT results in 20 of 130 patients (15%). Among these, unexpected or inconclusive lesions were found to be true recurrence or metastatic tumors in 4 patients, for whom surgery or chemotherapy was initiated based on PET/CT results. In 10 patients, lesions that were suspected as recurrence before PET/CT were regarded as negative because of lack of abnormal uptake, and scheduled treatment was cancelled. Initially planned chemotherapy regimen was altered in 6 patients according to their PET/CT scan results. To conclude, in 19 of 20 patients (95%) PET/CT had correctly managed therapy/follow-up plans according to the final diagnosis. However, the remaining one (5%) case with abnormal diagnostic CT scan was revealed to have lesion with no FDG uptake leading to cancellation of previously planned chemotherapy. The results of the changes in patients’ management are listed in Table 5.

## DISCUSSION

The optimal method for assessing early recurrence in patients with gastric cancer is unclear (18). Conventional imaging modalities (ultrasonography, CT and MRI) represent the standard for staging and restaging of gastric cancer (19,20). Conventional imaging is noninvasive and is the least costly of the available methods, although it has limited value in differentiating post-surgical changes from local tumor recurrence. Therefore, these techniques have limitations in terms of accurate assessment of recurrence (18,21). Compared with the large number of reports pertaining to PET or PET/CT findings of gastrointestinal tumors such as esophageal and colorectal cancer, there are only a few reports on PET and PET/CT findings of gastric cancer. However, their results were inconsistent. Jadvar et al. reported that FDG PET might be useful in the post-therapy evaluation of recurrent disease. Sim et al. and Park et al. also suggested that PET/CT might have a role of detecting recurrence in post-operative patients with gastric cancer. On the other hand, De Potter et al. reported that FDG PET might not be suitable as a primary tool for follow-up due to its moderate accuracy (14,15,16,22). In a recent study Baiocchi et al. concluded that oncological follow-up after radical surgery for gastric cancer should be based mainly on thoracoabdominal CT and 18-FDG-PET (23).

In our results, all values of diagnostic performance except for the specificity value were greater than 75%, and particularly the sensitivity value was up to 91% on a per-person basis. The lack of diagnostic spesificity in this study is thought to be especially due to low specificity value of locoregional recurrence, causing of false positive FDG uptake in anostomotic site which was the result of postoperational inflammatory changes. Another reason was the low specificity value of distant metastasis, especially caused by mediastinal lymph nodes. As CT part of the PET/CT scan was not diagnostic, it was not always possible to distinguish vascular structures from mediastinal lymph nodes. Also many metabolically active conditions like abdominal organs where physiologic uptakes are commonly found are the gastrointestinal and urinary tracts, can cause FDG uptake, thus can decrease the specificity of PET in detecting malignant lesions (22). These include the inflammatory lesions like granulamatous diseases, diverticulis and gastritis and the benign tumors like colonic adenomas (24,25). But these false positive conditions can be readily detected by side-by-side reviewing of anatomic correlation between PET and CT, thus improving spesificity and positive predictive value.

In some reports, it is mentioned that PET/CT has moderate sensitivity and specificity for detection of gastric cancer recurrence. Needless to say, false negative results mostly reflect their insufficient metabolic activity of a malignant lesion, but its small size can occasionally cause false negative interpretation, particularly in cases of peritoneal metastasis (16,26,27). Also it has been reported that SRC and mucinous adenocarcinoma showed significantly low FDG uptake and thus they can pose false-negative findings (28,29). However, our results were not consistent with these findings, although the number of patients in the SRC/mucinous adenocarcinoma subgroup was not large (n=25). In our results; in terms of whether or not histology had been SRC/musinous adenocarcinoma, although it was higher in the non SRC/mucinous adenocarcinoma group, the difference in diagnostic accuracy rates were not statistically significant on a per-person (84.7% vs. 72.0%) and per-lesion basis, except for liver metastasis.

It is reported that PET/CT influence patient management and decision making in the significant part of patients in the range of 14% to 52%. According to our results, after the integration of PET/CT scan in the patients’ follow-up, clinical management was changed in 20 (15%) of patients. Our results were thus competible with those in the literature for modification of treatment planning (2,30,31). In 19 of these 20 patients (95%), to our knowledge which is the highest per cent of accuracy ever reported, PET/CT had correctly-managed therapy/follow-up plans according to the final diagnosis.

Our study had several limitations. First, not all the recurred cases were confirmed by histopathological diagnosis. Therefore, there was the possibility of including cases in which false-positive lesions were treated as true-positive lesions by anti-cancer drugs, or true-positive lesions were not identified in the clinical setting. Secondly, 3-month interval may not be enough to confirm the absence of recurrence. In addition, the small sample size may have produced a statistically limited value. Another limitation was the retrospective design, so the suspicion of recurrence and proper indication of 18F-FDG PET/CT scans were not well defined. Because of this, we were unable to standardize the interval and methods of follow-up imaging studies and gastroscopy. This nature also could cause selection bias, because the patients who had not undergone PET/CT were excluded. Also, as the number of patients with advanced stage was high, our study population might be composed of patients who had a higher possibility of having recurrence.

Despite these deficiencies, our study has significance in giving us evidence of the role of fusion PET/CT in post-operative surveillance and in the clinical decision-making process. We suggest that PET/CT is highly useful in detecting or confirming recurrence once a patient with gastric cancer is clinically or radiologically suspicious of recurrence during follow-up. Moreover, our results together with those in the literature demostrate that integrated FDG PET/CT allows optimization of the treatment plan and might play an important role in decision making of treatment. Further well-designed prospective studies enrolling large populations are needed to establish the role of fusion PET/CT in detection of gastric cancer recurrence.

## CONCLUSION

In conclusion, in spite of several limitations and retrospective design, our results reveal that FDG PET/CT can provide useful information in discriminating true recurrence in patients with suspected gastric cancer recurrence and may have significant impact on clinical decisions/patient management in a considerable percentage of patients.

**Conflicts of Interest**

There are no conflicts of interest.

## Figures and Tables

**Table 1:**
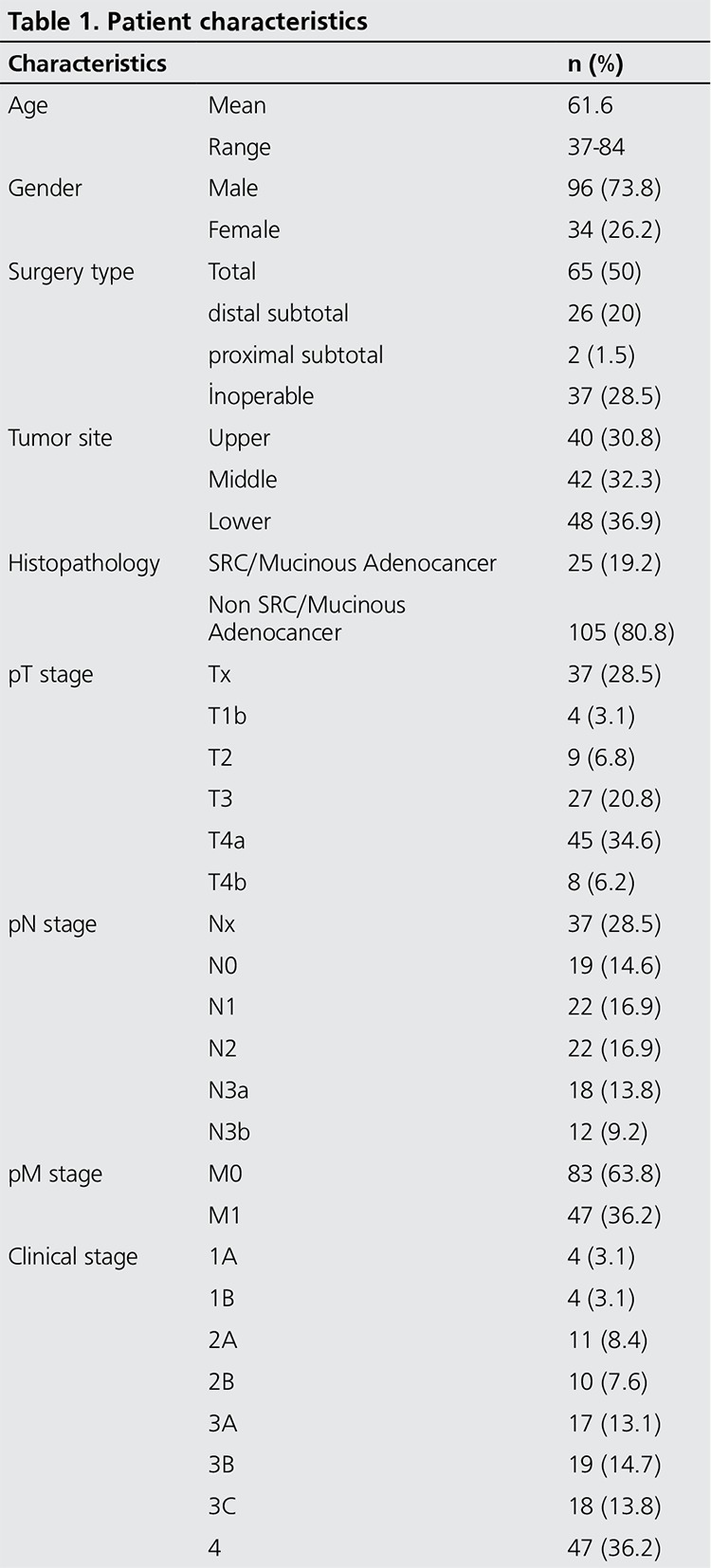
Patient characteristics

**Table 2:**
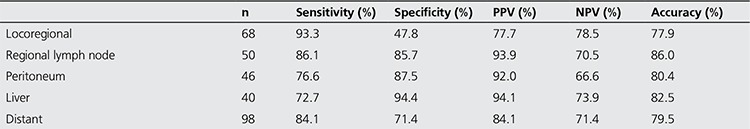
Diagnostic performance of PET/CT on the organ based analysis

**Table 3:**
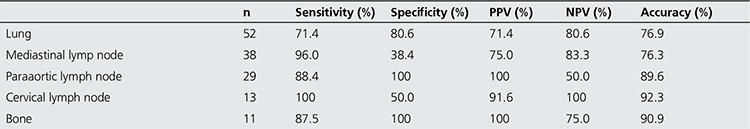
Diagnostic performance of PET/CT in the most common five distant metastasis sites when the distant metastasis group was evaluated separately

**Table 4:**
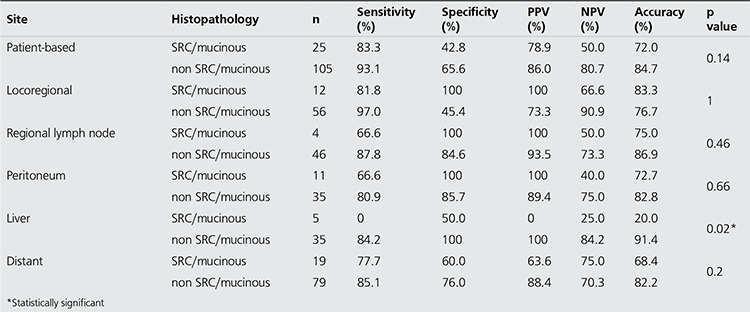
Diagnostic performance of PET/CT on the patient and organ based analysis in terms of whether or not histology had been SRC/musinous adenocarcinoma

**Table 5:**
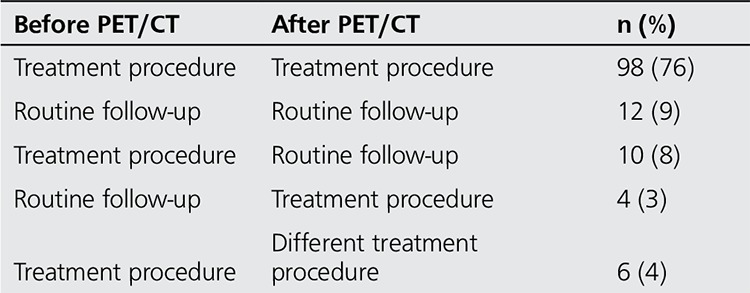
Impact of PET/CT in clinical decision making
